# Persistently erected penis in a child for 6 months: A management dilemma

**DOI:** 10.4103/0971-9261.42573

**Published:** 2008

**Authors:** Sukanta Das, Dipak Ghosh, Akhilesh Agarwal, Suranjan Haldar

**Affiliations:** Department of Pediatric Surgery, General Surgery Medical College, Kolkata, India

**Keywords:** Malignancy idiopathic, priapism

## Abstract

Priapism is the presence of a persistent, usually painful, erection of the penis unrelated to sexual stimulation or desire. It is a true emergency requiring urgent intervention. Priapism is frequently idiopathic in etiology, but it is associated with a number of important medical conditions and pharmacologic agents. Cases have been reported in world literature on children having priapism, the etiology of these cases are mostly hematological. Our case is a child having persistently erected penis for more than 6 months. Despite a thorough search, no report of similar case could be found in world literature.

## INTRODUCTION

Priapism (Greek πριαπισμóς, the erection) is a painful and potentially harmful medical condition in which the erect penis (erection) does not return to its flaccid state (despite the absence of both physical and psychological stimulation) within 4 h.

It associated with many medical and surgical morbidities, namely, leukemia, Fabry's disease, hematological disorders (sickle cell disease) and pelvic trauma. It is an emergency as this is an extremely painful condition, and if the treatment is not administered immediately, it may lead to irreversible complications such as complete necrosis.

## CASE REPORT

**Figure 1:**
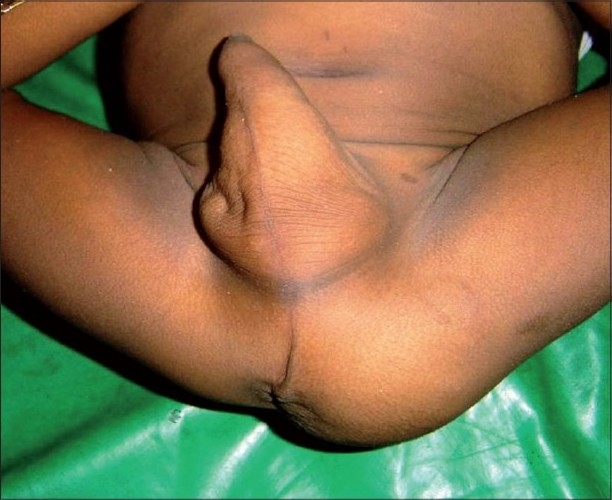
Erected penis with gluteal mass

A 3.5-year-old boy presented with a history of persistently erected penis for 6 months [Figure 1]. As stated by his mother, it started insidiously. There was no history of trauma.

He had a lump in left gluteal region for 6 months. He suffered with occasional local pain and increase in the body temperature. There is no history of any bleeding from gums, easy bruisbility, bleeding per rectum, etc., which is suggestive of blood dyscrasias. There is no history of drug intake for long time. There is no family history of syphilis.

**Figure 2:**
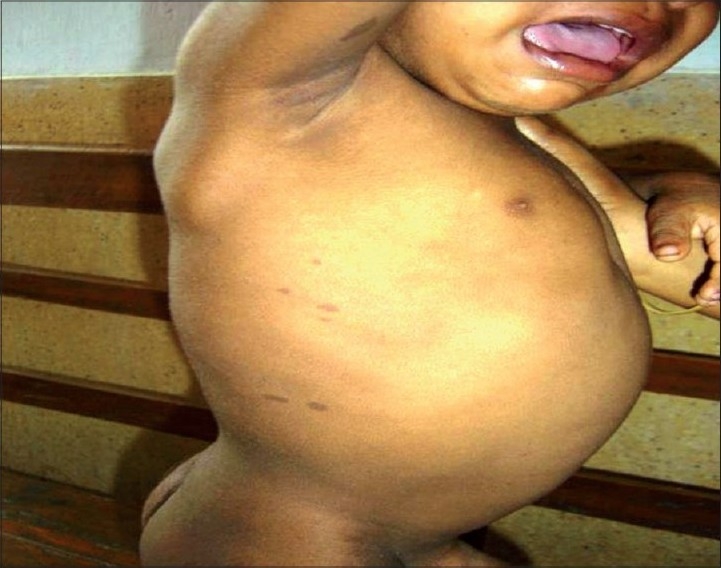
café-au-lait spots on trunk

There is a mass at the left gluteal region that extends till the base of the penis. It is tender and noncompressible. There is no local rise in the body temperature. There is patchy pigmentation over the trunk and back [Figure 2]. There is no palpable lump in the abdomen.

**Figure 3:**
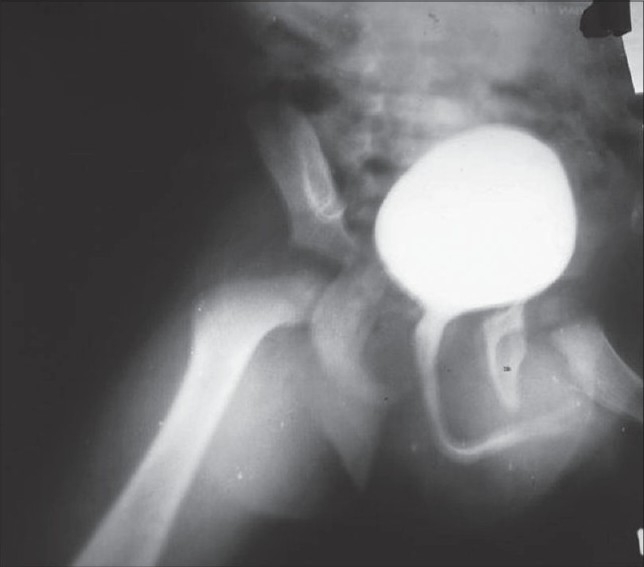
Voiding cystourethrogram showing elongated post urethra

**Figure 4:**
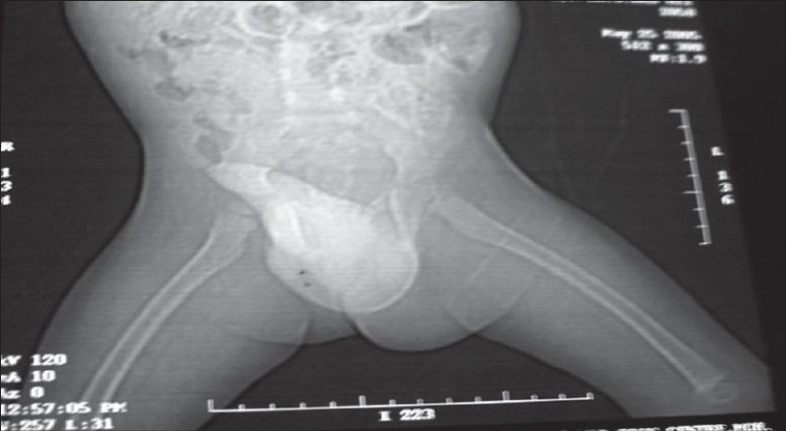
CT reconstruction showing the mass

Complete hemogram showed no abnormalities. Ultrasonography of the entire abdomen showed no abnormalities, which is suggestive of any pelvic mass. Aspiration cytology of the gluteal mass showed granular necrotic material with RBC. Voiding cystourethrogram showed elongated and narrowed posterior urethra [Figure 3]. CECT of pelvis showed areas of enhancement that merged with the left half of the penis base and the left gluteal region [Figure 4]. No obvious calcification was observed. Color Doppler showed no abnormality in the blood flow. After all these investigations, a surgical intervention was planned in order to excise the mass. An “S”-shaped incision was given starting from left gluteal cleft to the base of the penis. A well-circumscribed mass was found in the left gluteal region that extended to the base of penis. The mass was excised from the gluteal region. At the base of penis, the mass was found encasing the left corpora cavernosa and was also found pressing on the right corpora. The corpora spongiosa was free. Both corpora cavernosa and corpora spongiosa were freed from the mass. On clinical examination, there was no fibrosis at the region. At the region of encasement, a fine needle was pricked and there was free flow of blood.

**Figure 5:**
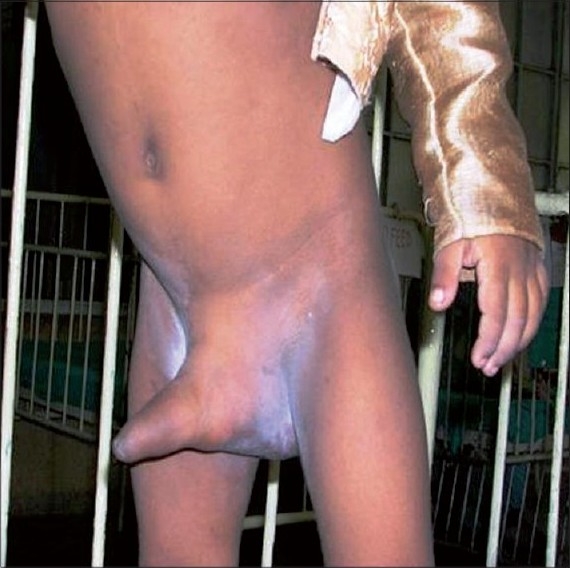
Photograph taken 1½ years after the surgery

The wound was closed. Postoperatively, there was seroma formation at the gluteal region, which was drained. The histopathology came out to be lipofibroma. One and a half year after the surgery, the child still presented with the erection; however, it was painless. No recurrence in the gluteal region was seen [Figure 5].

## DISCUSSION

Priapism is a painful and potentially harmful medical condition in which the erect penis does not return to its flaccid state despite the absence of both physical and psychological stimulation.[1] There are various etiologies for priapism, ranging from benign to malignant ones. Children with priapism are typically those who have leukemia, sickle-cell disease and trauma either to the penis or to the area underneath the penis, the perineum. In addition, spinal cord injuries can cause priapism. Extremely rare causes of priapism include drug side effects, but typically these drugs are not used in children.[2] Priapism is an emergency demanding prompt medical attention.[3] If a patient gets treatment within 4-6 h, the erection can almost always be reduced with medication. The first step for the patient with priapism with less than 4 h duration is the use of decongestant medication such as pseudoephedrine and terbutaline, which may decrease the blood flow to the penis and is very successful in early cases.[4] If the erection does not respond, then aspiration is performed. The longer the condition goes without treatment, the worser will be the prognosis. If at all, the above mentioned treatment fails to relive the patient, then a saphenocavernous shunt can be carried out. But the benefits of this shunt are still questionable.

Complications can occur after the treatment of priapism. They are as follows: recurrence of priapism, impotency[5] infections, skin necrosis, infection of the corporal body, damage and strictures to urethra and pulmonary embolism - in rare cases.

## References

[1] Van Der Horst C, Stuebinger Henrik, Seif Christoph, Melchior Diethild, Martínez-Portillo FJ, Juenemann KP (2003). Priapism: Etiology, pathophysiology and management. Int Braz J Urol.

[2] Beers MH, Berkow R (1999). The Merck Manual of Diagnosis and Therapy.

[3] Harmon WJ, Nehra A (1997). Priapism: Diagnosis and management. Mayo Clin Proc.

[4] Cherian J, Rao AR, Thwaini A, Kapasi F, Shergill IS, Samman R (2006). Medical and surgical management of priapism. Postgrad Med J.

[5] Emond AM, Holman R, Hayes RJ, Serjeant GR (1980). Priapism and impotence in homozygous sickle cell disease. Arch Intern Med.

